# The Origin of Alcohol

**Published:** 1902-08

**Authors:** W. B. Parks

**Affiliations:** Atlanta, Ga.


ATLANTA

### Journal-Record of Medicine.

Successor to Atlanta Medical and Surgical Journal, Established 1855, and Southern Medical Record, Established 1870.

VOL. IV. AUGUST, 1902. NO. 5.

BERNARD WOLFF, M.D., EDITOR, Nos. 319-20 Prudential.

Published Monthly.

M. B. HUTCHINS, M.D., BUSINESS MANAGER, No. 64 Marietta St.

### *ORIGINAL COMMUNICATIONS.*

### THE ORIGIN OF ALCOHOL.*

BEING PREHISTORIC, ITS SUPPOSED VALUE AS A BEVERAGE AND A MEDICINE IS INFLUENCED BY SUPERSTITION AND IGNORANCE.

BY W. B. PARKS, M.D.,
ATLANTA, GA.

I fully realize that the above statement is a broad assertion, and the only apology I have to offer is that it is a lamentable fact that it was not uttered and proved ages ago, thereby saving the necessity and embarrassment of convincing an intelligent people that they had been hoodwinked by superstition and ignorance into the universal belief that alcohol possesses great value as a beverage, and is indispensable as a medicine.

We have only to refer to a prehistoric people, whose intelligence

*Read before the Georgia Sociological Society, Atlanta, Ga., June 24, 1902.

was that of a savage, to find that they accidentally discovered that the juices of tropical fruits had undergone fermentation in the cup-shaped palm leaves, into which they had dropped, and when they drank it in this state it produced intoxication, leading these unenlightened people to suppose that this delightful sensation, which seemed harmless to them, was not only a cure for their simple ailments, but made them stronger and more fearless in the terrible wars which they waged in defense of their hunting-grounds.

We have the best evidence to show that through a long line of heredity we have handed down to us from those prehistoric savage people the belief, inspired by superstition and ignorance, that alcohol is to-day a panacea for most of our ills, and will serve our purpose in all conditions; but, thank God, blind superstition is being removed by the effulgent light of the Christian religion. Science and progress are fast displacing the inexcusable ignorance which has been so well fortified by arrogant bigotry, and the day is not far distant when King Alcohol, with all his allied forces, will be returned to his Satanic Majesty, with compound interest of misery, death and destruction.

We will now examine the absurd inconsistencies in the use of alcohol which tradition has handed down to us.

When a new heir made his advent in a home the occasion was, and is, often celebrated by the father plunging into intoxication in the belief that it would increase his joy. On the other hand, a year later, if the gray monster, Death, should claim this promising boy, the father would seek to drown his grief in beastly drunkenness. If the grief is made less by intoxication, why is not the joy made less by the same?

Again, if this same father finds himself facing a freezing blizzard, far away from his cozy home, he tries to warm himself by the benumbing effect of liquor. A few months later, as he quickens his footsteps on his return home, the intensity of the hot sun seems to increase, and he thinks that if he will again benumb himself with an intoxicant he will be cool and comfortable.

We might also see him on his return informing his good wife of the prospects of a fortune in the near future, and as her joy and approval thrill him with happiness, he flies to his bottle to increase his pleasure. But a telegram comes, blighting his hopes with the news that his long journey was in vain, and the expected fortune becomes a dream of the past. Again he seeks consolation and strength in the bottle. If intoxication increased his happiness when he expected a fortune, how could it remedy his trouble and disappointment? Later, while in the best of health, he stupefies himself with liquor that he may feel better. Then he is stricken with disease, and he thinks that intoxication will relieve his pains.

If intoxication made him feel better when he was well, why should it not, when he was sick, make him feel worse?

These inconsistencies in the use of alcohol are not only believed, but they have been practiced for hundreds of years, from the time when our Anglo-Saxon ancestors believed that heaven was a place where they would pass their time drinking strong liquors from the skulls of their foes slain in battle, down to this enlightened twentieth century, when we still find alcohol reckoned of great value in the commercial world, and considered an indispensable factor in politics and a ready remedy in all kinds of sickness, all of which we may say is the direct result of a stigmatized taint of superstition and inexcusable, arrogant bigotry and ignorance.

In the face of all this, the good people along the lines of time and progress have inaugurated ways and means of enlightening mankind by organizing various temperance movements; by requiring the applicants for membership in many church organizations to renounce alcohol as an emissary of the devil, and by high license, police restrictions and prohibition laws indorsed by a vote of the people. But all of these, including local option, have failed in a great measure to eradicate the deep-rooted suggestion that alcohol is necessary to mankind, and I am sorry to say that, although it came to us from a benighted people, this Satanic belief has fastened its hold upon the educated, and even upon many ministers of the gospel, who contend that they have a right to use alcohol for medicinal purposes.

It becomes our solemn duty to ferret out the cause of the slow headway made against alcohol. We might first examine the moral methods, and it will be seen that they appeal largely to the sentiment of man. A great temperance movement sweeps the country like a tidal wave, then the enthusiasm gradually subsides, and a lethargy follows in its wake seeming to reestablish King Alcohol on his throne with increased power. Then, another method is resorted to, the strong arm of the law, and who shall dare to question its power to control a recognized national evil? Yet, from past experience we find that this method awakens in man a spirit of resentment, and he repeals the law on the ground of personal liberty, and from the cry of personal liberty factions are born, the leaders of which propose to rescue the country by putting into their platform a prohibition plank. Then the local option faction raises the cry that such a plank would forever cover up all that local option has done, and as these complicated emergencies arise we find father voting against son; the ministers writing and speaking against one another, and such a turmoil of political wrangle that, in the words of a certain statesman, we can hear them asking: "Where are we at?"

While this is going on the cause of alcohol is fortifying against another attack.

Inasmuch as the sentimental method is transitory and unstable in the end, and the law fails to enforce prohibitory measures, and local option is only practicable in thinly populated districts, utterly failing in the large cities where it is greatly needed, it behooves us to study the situation earnestly and carefully and try to ascertain if there is a method by which the stronghold of King Alcohol can be overthown.

Our present stage of progress and development requires means and methods not applicable years ago, when the effects of alcohol began to be recognized as a direful curse to mankind. Man has reached such a high state of mental development that he is disposed to reason out difficult problems, for experience teaches him that, while calmly exercising his highest and best reasoning mental faculties, he approaches nearer the citadel of the soul, thereby receiving the benefit of God-given intuitive knowledge.

Hence, I insist that we have reached the stage of the educative method by which the effects of the dreaded monster alcohol can be analyzed, not from a collective or rational standpoint, but by the aid of science and chemistry we are enabled to demonstrate the poisonous effects of alcohol in all parts of the human organism.

To elucidate—It has been demonstrated that even small doses of alcohol coagulate the albumen in the blood: it corrugates or shrinks up the fibrous tissue, which renders the system less resistive to all diseases. It is no longer given in surgical shocks, or shock from other injuries, for the reason that it paralyzes the blood-vessels. These vessels, especially the arteries, are a continuation of the heart tissue, receiving their nervous impulse from the same nerves which supply the heart, all of which are given off from the central nervous system. Therefore, no substance can paralyze the blood-vessels without also paralyzing the heart. On the same principle, alcohol does not warm the system when suffering from cold in winter, because it paralyzes the nerves of the heart and blood-vessels, thereby causing the blood to remain too long in the extremities, or too long from the heart, which condition can cause heart-failure.

As to the food value of alcohol, by its oxidizing process when taken into the system you gain in measure one ounce of energy, but you lose two ounces of material, or fuel, thus losing twice as much as you gain.

Without showing further the great progress of the study of the effects of alcohol upon the human system, we are ready to prove that alcohol is a curse as a beverage, and is delusive as a medicine, misleading in its effects both physician and patient, and we would recommend that its evil effects be taught in all of our schools, and also taught and demonstrated from the lecture platforms, the pulpits, and in our Sunday-schools, vigorously and persistently, without apology or compromise, until the traditional, ignorant and superstitious beliefs are forever eradicated from the minds of the young.

And while this is being done, we must not fail to sound another note of warning, for like those prehistoric people who innocently used the poisonous alcohol which they found in the cup-shaped palm leaves, we have innocently fallen into the habit of using another poison, not found however on the open palm leaf, in the form of fermented fruit juices, for in this age of progress such a beverage would not tempt any one; but we have a poison which successfully secreted itself in the very fiber of the cocoa leaf, and it has insidiously won favor with the young, the fair sex, and with all, in the shape of a cool, refreshing drink—not in the grog shop, but at the soda fount, and even in small, cheap bottles which any child can buy at our fruit stands and provision stores.

While I appreciate the fact that I will be criticized for sounding this note of warning, God forbid that I should fail to do my duty, for the very poison that is hidden in the sparkling glass of the delightful drink at the soda fountain will, if not checked, fasten its hold upon the innocent, until a few generations later, when its hereditary effect will begin to show itself, our descendants will be idiotic in mind and dwarfs in physical stature—a race of people utterly unfit for the mission of human beings on the earth.

I need say no more. Surely I have said enough to make it evident that this great question deserves the careful and conscientious attention of every wise and good man who loves his fellows and desires to uplift them, and save their souls and bodies. You must get rid of "King Alcohol," or he will destroy you or some loved one in your family. Compromise with this agent of the devil means his final supremacy.

